# Carbohydrate Hydrolytic Potential and Redundancy of an Anaerobic Digestion Microbiome Exposed to Acidosis, as Uncovered by Metagenomics

**DOI:** 10.1128/AEM.00895-19

**Published:** 2019-07-18

**Authors:** Marie Bertucci, Magdalena Calusinska, Xavier Goux, Corinne Rouland-Lefèvre, Boris Untereiner, Pau Ferrer, Patrick A. Gerin, Philippe Delfosse

**Affiliations:** a Environmental Research and Innovation Department, Luxembourg Institute of Science and Technology, Belvaux, Luxembourg; b Laboratory of Bioengineering, Earth and Life Institute, Applied Microbiology, Université Catholique de Louvain, Louvain-la-Neuve, Belgium; c Institute of Ecology and Environmental Sciences, Research Institute Development, Sorbonne Universités, Bondy, France; University of Tartu

**Keywords:** *Bacteroidetes*, biotechnology, enzymes, molecular biology, polysaccharides, recombinant-protein production

## Abstract

Increased hydrolysis of easily digestible biomass may lead to acidosis of anaerobic reactors and decreased methane production. Previously, it was shown that the structure of microbial communities changed during acidosis; however, once the conditions are back to optimal, biogas (initially CO_2_) production quickly restarts. This suggests the retention of the community functional redundancy during the process failure. In this study, with the use of metagenomics and downstream bioinformatics analyses, we characterize the carbohydrate hydrolytic potential of the microbial community, with a special focus on acidosis. To that purpose, carbohydrate-active enzymes were identified, and to further link the community hydrolytic potential with key microbes, bacterial genomes were reconstructed. In addition, we characterized biochemically the specificity and activity of selected enzymes, thus verifying the accuracy of the *in silico* predictions. The results confirm the retention of the community hydrolytic potential during acidosis and indicate *Bacteroidetes* to be largely involved in biomass degradation. *Bacteroidetes* showed higher diversity and genomic content of carbohydrate hydrolytic enzymes that might favor the dominance of this phylum over other bacteria in some anaerobic reactors. The combination of bioinformatic analyses and activity tests enabled us to propose a model of acetylated glucomannan degradation by *Bacteroidetes*.

**IMPORTANCE** The enzymatic hydrolysis of lignocellulosic biomass is mainly driven by the action of carbohydrate-active enzymes. By characterizing the gene profiles at the different stages of the anaerobic digestion experiment, we showed that the microbiome retains its hydrolytic functional redundancy even during severe acidosis, despite significant changes in taxonomic composition. By analyzing reconstructed bacterial genomes, we demonstrate that *Bacteroidetes* hydrolytic gene diversity likely favors the abundance of this phylum in some anaerobic digestion systems. Further, we observe genetic redundancy within the *Bacteroidetes* group, which accounts for the preserved hydrolytic potential during acidosis. This work also uncovers new polysaccharide utilization loci involved in the deconstruction of various biomasses and proposes the model of acetylated glucomannan degradation by *Bacteroidetes*. Acetylated glucomannan-enriched biomass is a common substrate for many industries, including pulp and paper production. Using naturally evolved cocktails of enzymes for biomass pretreatment could be an interesting alternative to the commonly used chemical pretreatments.

## Abstract (precis)

The enzymatic hydrolysis of lignocellulosic biomass is mainly driven by the action of carbohydrate-active enzymes. By characterizing the gene profiles at the different stages of the anaerobic digestion experiment, we showed that the microbiome retains its hydrolytic functional redundancy even during severe acidosis, despite significant changes in taxonomic composition. By analyzing reconstructed bacterial genomes, we demonstrate that *Bacteroidetes* hydrolytic gene diversity likely favors the abundance of this phylum in some anaerobic digestion systems. Further, we observe genetic redundancy within the *Bacteroidetes* group, which accounts for the preserved hydrolytic potential during acidosis. This work also uncovers new polysaccharide utilization loci involved in the deconstruction of various biomasses and proposes the model of acetylated glucomannan degradation by *Bacteroidetes*. Acetylated glucomannan-enriched biomass is a common substrate for many industries, including pulp and paper production. Using naturally evolved cocktails of enzymes for biomass pretreatment could be an interesting alternative to the commonly used chemical pretreatments.

## INTRODUCTION

Anaerobic digestion (AD) of biomass (including biowaste) is a process aiming at producing biogas, i.e., a mixture of methane (CH_4_), carbon dioxide (CO_2_), and trace gases (i.e., H_2_, NH_3_, and H_2_S) (1). Biogas is a result of the joint action of diverse microorganisms that act synergistically to decompose organic matter. In the first stage, namely hydrolysis, the organic matter (i.e., fats, proteins, and polysaccharides) is deconstructed by fermentative bacteria into soluble molecules. During the acidogenesis and acetogenesis stages, bacterial consortia convert the resulting monomers and oligomers mainly into volatile fatty acids (VFAs), including acetate, CO_2_, and H_2_. Finally, methane is produced by methanogenic archaea during the methanogenesis stage (2). Even though different environmental factors and operational conditions can lead to the dysfunctioning of anaerobic digesters, acidification caused by an accumulation of VFAs (here, referred to as acidosis) is one of the most often recorded phenomena (3, 4). It results from the decoupling of the hydrolytic and acidogenic stages (which perform too fast due to, e.g., higher multiplication rates of involved organisms under specific conditions) from the downstream acetogenesis and methanogenesis stages (which are too slow due to, e.g., slower multiplication rates of the acetogenic bacteria and methanogenic archaea) (5). As reported from previous studies, acidosis leads to a change in the microbial community structure, i.e., the taxonomic composition (4, 6). Both bacterial and archaeal communities are affected, and, as a result, methane production is slowed down and sometimes interrupted. Interestingly, following a recovery stage, the newly established community relatively quickly restarts the biogas production (3, 4). This suggests that the functional community redundancy (similar metabolic functions performed by distinct coexisting microorganisms) (7) occurs in AD during the acidosis.

Hydrolysis is often considered a bottleneck of the AD process because it either underperforms (in the case of the recalcitrant biomass, e.g., highly lignified or high crystalline cellulose content) or outperforms (fast hydrolysis of an easily digestible biomass can lead to acidosis). The enzymatic hydrolysis of lignocellulose biomass is mainly driven by the action of carbohydrate active enzymes (CAZymes) and especially by glycoside hydrolases (GHs), carbohydrate esterases (CEs), polysaccharide lyases (PLs), and other auxiliary enzymes (AAs) (8). Carbohydrate-binding modules (CBMs) are noncatalytic contiguous amino acid sequences, which bind hydrolytic enzymes to their carbohydrate substrates. They usually exist as modules within larger enzymes, but some can be independent proteins (9). Glycosyltransferases (GTs) are also classified within the CAZy database as they are involved in the biosynthesis of glycosidic bonds from phosphate-activated sugars donors. GHs hydrolyze and/or transglycosylate the glycosidic bonds, CEs hydrolyze ester bonds, and AAs are redox enzymes acting in conjunction with other CAZymes, many of which are involved in lignin degradation. PLs cleave bonds from uronic acid-containing polysaccharide chains (nonhydrolytic cleavage of glycosidic bonds). With the emergence of the high-throughput sequencing technologies, owing to the reduced cost and easier access, researchers have investigated the metabolic potential (referring to the gene content) of the microbial communities present in anaerobic environments. In a variety of studies, microbial communities of full-scale and lab-scale anaerobic digesters under optimal (10) and dysfunctioning conditions have been characterized (4, 11–13). Among bacteria, *Bacteroidetes* and *Firmicutes* have often been cited as the most dominant phyla, and their genomes were shown to contain high number of CAZyme-coding genes (14–16). CAZyme-coding genes can be expressed separately in bacterial genomes or they can form operons of coexpressed genes, e.g., cellulosomes in *Firmicutes* or polysaccharide-utilization loci (PULs) in *Bacteroidetes*. While cellulosomes have been given large scientific attention in the past (17, 18), PULs were much less investigated (17, 19). PULs are defined as gene clusters encoding cell envelope-associated enzymes that allow *Bacteroidetes* to bind to and degrade a specific polysaccharide and to import the released oligosaccharides inside the cell (20, 21). They are composed of at least one pair of *susC* and *susD* genes encoding a TonB-dependent transporter (TBDT) and a cell-surface glycan-binding protein (SGBP), respectively (22). The protein SGBP is involved in the binding of carbohydrates while TBDT transports them through the outer membrane. The first PUL was identified from Bacteroides thetaiotaomicron isolated from the human gut and dedicated to starch degradation (23). During the last decades, a number of PULs have been discovered from omics studies, unravelling a wider diversity of CAZyme-coding genes than previously thought (24–28). One study reported the characterization of three PULs (two targeting xylan and one targeting pectins) using combined omics approaches, i.e., transcriptomics, proteomics and metabolomics, and originating from the marine environment (29). Another study identified a xyloglucan-specific PUL (from the human gut) through a metagenomics approach that was further experimentally validated (30).

The general purpose of the present study was to characterize the hydrolytic potential of the microbial community in an anaerobic digestion reactor. In particular, we aimed at assessing the carbohydrate hydrolytic potential in the context of an acidosis event, which is one common type of reactor dysfunction. Previous studies have shown that acidosis impacts microbial community structure at the taxonomic level. Here, we wanted to evaluate the community functional redundancy, with a special focus on the carbohydrate hydrolytic capacity of the anaerobic digester community. Metagenomic reconstruction allowed identification of 4,148 genes putatively assigned to CAZy families (including 49 putative PULs); only 1,052 of these were further assigned an Enzyme Commission (EC) class related to a potential hydrolytic function. To further link the community hydrolytic potential with key microbes, we investigated the newly reconstructed metagenome-assembled genomes (MAGs). Additional enzymatic activity assays were performed on a subset of predicted CAZymes belonging to a PUL named PUL219 from a dominant *Bacteroidetes* MAG in order to confirm their predicted activities.

## RESULTS

### Functional redundancy of hydrolytic metabolism under changing environmental conditions in AD reactors.

**FIG 1:**
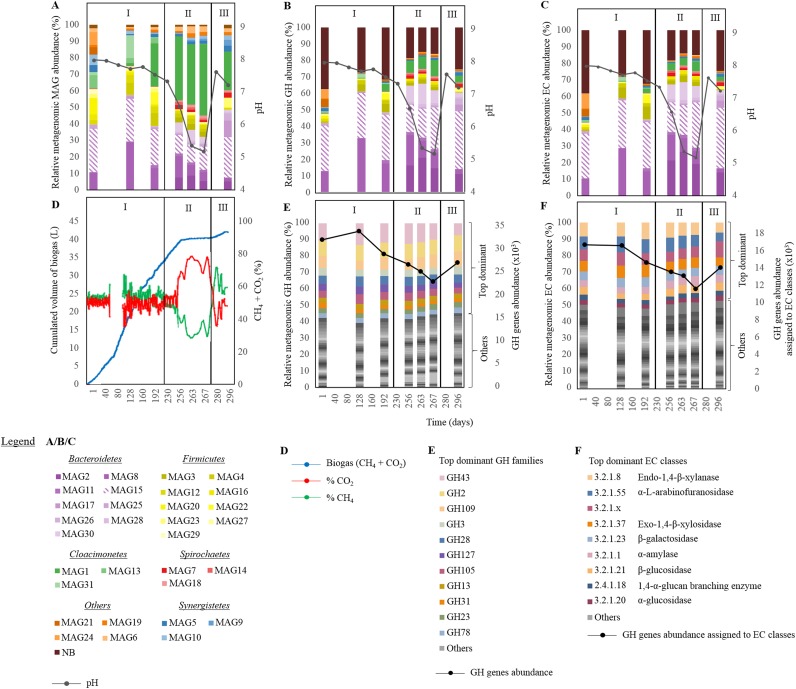
Metagenomics-assisted characterization of microbes and their hydrolytic potential throughout the anaerobic digestion (AD) experiment. (A) Relative metagenomic abundance of the different metagenome-assembled genomes (MAGs). (B) Relative metagenomic abundance of the different glycoside hydrolases (GH) colored according to the reconstructed MAG. (C) Relative metagenomic abundance of the different GHs additionally assigned to an Enzyme Commission (EC) category, colored according to the reconstructed MAG. (D) Characteristics of the biogas produced, including the cumulative biogas volume and the percentage of CH_4_ and CO_2_ in the biogas at the different stages of the experiment. (E) Relative metagenomic abundance of the different GHs colored according to the GH family. The top dominant families further discussed in the manuscript are highlighted. GH gene abundance, at the microbiome (whole-community) level, is represented by a black solid line. (F) Relative metagenomic abundance of the different GHs additionally assigned to an EC category, colored according to the EC category. The top dominant categories further discussed in the manuscript are highlighted. GH gene abundance assigned to EC classes is represented by a black solid line. Roman numerals indicate phases I to III of the AD experiment, as follows: I, decreasing hydraulic retention time (HRT) and increasing organic loading rate (OLR) of sugar beet pulp; II, acidosis; III, process recovery. NB, not binned.

**FIG 2:**
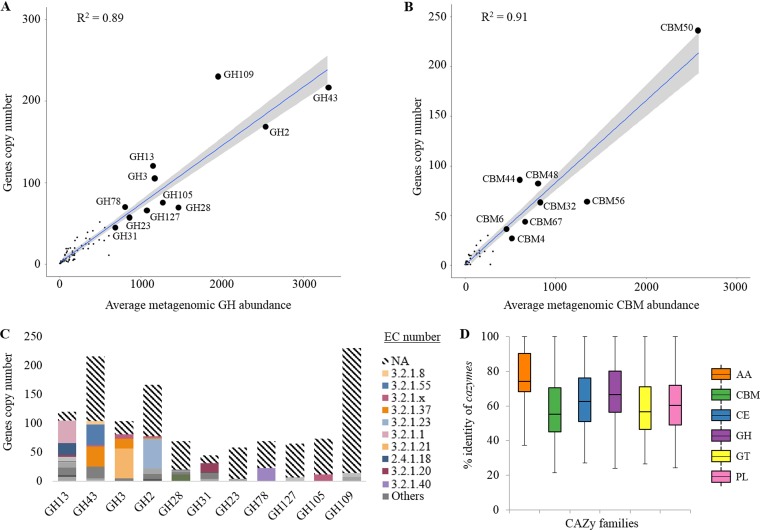
Characteristics of CAZy-coding genes identified in the anaerobic digestion (AD) reactor. (A) Correlation between the average metagenomic glycoside hydrolase (GH) abundance and the absolute number of gene copies at the microbiome (whole-community) level. Top dominant families further discussed in the manuscript are highlighted. (B) Correlation between the average metagenomic carbohydrate binding module (CBM) abundance and the absolute number of gene copies at the microbiome level. Top dominant CBM families are highlighted. (C) Assignment of the top dominant GHs to an Enzyme Commission (EC) category. NA, not assigned; EC 3.2.1.8, endo-1,4-β-xylanase; EC 3.2.1.55, α-l-arabinofuranosidase; EC 3.2.1.37, β-xylosidase; EC 3.2.1.23, β-galactosidase; EC 3.2.1.1, α-amylase; EC 3.2.1.21, β-glucosidase; EC 2.4.1.18, 1,4-α-glucan branching enzyme; EC 3.2.1.20, α-glucosidase; EC 3.2.1.40, α-l-rhamnosidase. (D) Box plot representations of the percentage identity of the CAZymes to proteins in the NCBI nonredundant protein database. The different CAZy families represented include auxiliary enzymes (AA), carbohydrate-binding modules (CBM), carbohydrate esterases (CE), glycoside hydrolases (GH), glycosyltransferases (GT), and polysaccharide lyases (PL).

In our previous study (4), the microbial composition of the community dynamics was investigated with the use of high-throughput 16S rRNA gene amplicon sequencing in triplicate lab-scale AD reactors operated for 300 days. Reactors were sequentially exposed to a decreasing hydraulic retention time (HRT) and increasing organic loading rate (OLR; phase I) of dry sugar beet pulp, leading to acidosis (phase II) and process recovery (phase III), as indicated in Fig. 1. Here, the hydrolytic potential of the microbial community from one of the reactors (named R3 in Goux et al. [4]) was further investigated with the use of metagenomics. Genomic DNA was extracted from seven samples taken at different stages of the experiment and sequenced using a high-throughput approach (31). As a result, over 30,000 contigs were reconstructed. Out of the 75,002 nonredundant protein coding genes identified in the reconstructed contigs, 4,148 (representing 5.53% of all coding sequences) were assigned to CAZy families (dbCAN analysis) (32), representing GH, GT, AA, CE, and PL, as well as CBM classes. In total, 1,052 putative CAZymes were further functionally classified into EC protein categories (Hotpep analysis) (33). At the microbiome level, we found a good correlation between the average metagenomic GH coding gene abundance at the different stages of the experiment and their gene copy numbers (*R*^2^ = 0.89, *P* < 0.001) (Fig. 2A).

Selective pressure applied to the reactor led to a pH drop, and a significant variation over time in the abundance of specific microorganisms (bacterial MAGs) was observed (chi-square test *P* value, <0.001) (Fig. 1A to C). At the same time, the community gene profiles were conserved (based on the profile of the clusters of orthologous groups, or COGs [34]) (see Fig. S1A in the supplemental material), as well as acetogenesis and methanogenesis pathways (based on the profile of the Kyoto Encyclopedia of Genes and Genomes (KEGG) orthology [KO]) (35) (Fig. S1B and C), including during the acidosis stage and showing the community functional redundancy. The hydrolytic potential of the whole community (separately investigated based on the GH family profile and the EC profile) was also maintained during the whole AD experiment, despite a slight decrease of the abundance of genes coding for hydrolytic enzymes during the acidosis stage (Fig. 1E and F; Fig. S1D and E). Three GH families were overrepresented (based on their metagenomic abundances and numbers of the different genes present) (Fig. 1 and 2), including GH43 (average metagenomic abundance of 5.6% ± 1.1% of the total CAZyme gene content), GH2 (4.3% ± 0.3%), and GH109 (3.4% ± 0.5%). Other families such as GH28 (2.5% ± 0.3%), GH105 (2.1% ± 0.4%), GH3 (2.0% ± 2.0%), and GH13 (2.0% ± 0.2%) were also well represented. Despite their identical CAZy family annotations, different hydrolytic activities (EC category) were assigned to proteins representing a single CAZy family (Fig. 2C). Regarding their average metagenomic abundance, the hydrolytic portion of the metagenome was dominated by EC 3.2.1.8 (endo-1,4-β-xylanase; 8.2% ± 1.3% of all CAZyme-coding gene assigned to an EC category), EC 3.2.1.55 (α-l-arabinofuranosidase; 7.7% ± 4.4%), EC 3.2.1.37 (exo-1,4-β-xylosidase; 6.5% ± 0.8%), and EC 3.2.1.23 (β-galactosidase; 5.3% ± 0.5%) (Fig. 1F). Similar to abundance of GHs, the average metagenomic abundance of CBM coding genes was highly correlated to the total gene copy numbers in the microbiome (*R*^2^ = 0.91, *P* < 0.001) (Fig. 2B). The set of putative CBMs fell into 36 families (Fig. S1E), with CBM50 being the most abundant. In total, 91 coding genes from CBM50 were identified, and 20.9% of them were associated with a GH domain in a single protein.

Out of the identified CAZyme genes, 65.3% were further assigned to the different reconstructed MAGs. BLAST analysis against the NCBI nonredundant protein database showed overall low identities of the identified CAZymes for most of the MAGs (Fig. 2D). Roughly, 13.5% of predicted CAZymes showed high similarity (≥95%) to the entries in the nonredundant protein database. This confirms the novelty of the novel enzymes. AA class seemed more conserved (higher sequence similarity), as previously demonstrated for the cow rumen metagenome (36). Interestingly, around 1.7% of all CAZyme-coding genes were annotated as AAs. The AA genomic content of *Spirochaetes* was much higher than that for the other MAGs (5.4% ± 2.9% of the CAZyme-coding genes versus 2.5% ± 1.9% for *Firmicutes* and 0.6% ± 0.5% for *Bacteroidetes*).

### *Bacteroidetes* may be favored in some anaerobic digesters owing to its polysaccharide hydrolytic potential as reflected by higher CAZy diversity and genomic content.

**FIG 3:**
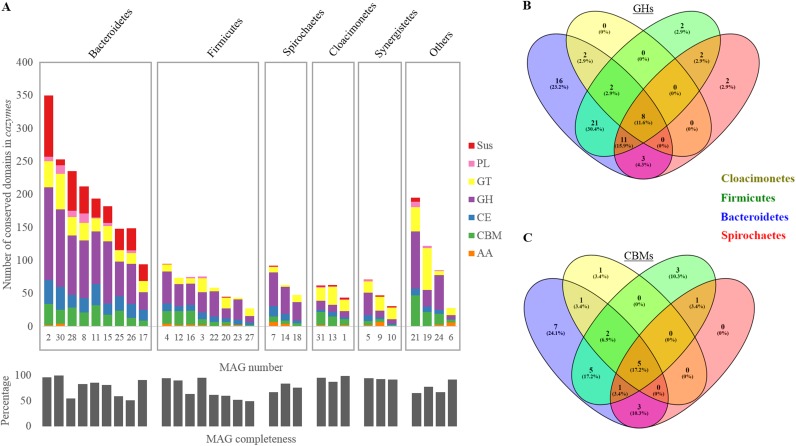
Characterization of the CAZy-coding genes (*cazymes*), including *sus*-like genes, in the reconstructed MAGs. (A) Distribution of the different conserved domains assigned to CAZy-coding genes in the reconstructed MAGs. (B and C) Venn diagram representation of the shared diversity of GHs and CBMs in MAGs assigned to the four main bacterial phyla present in the reactor. For the description of MAGs, refer to Table S1 in the supplemental material. The different CAZy families represented include auxiliary enzymes (AA), carbohydrate-binding modules (CBM), carbohydrate esterases (CE), glycoside hydrolases (GH), glycosyltransferases (GT), and polysaccharide lyases (PL).

We found that representatives of the phylum *Bacteroidetes* were among the most abundant MAGs across the digestion experiment (average metagenomic read abundance, 41.9% ± 8.4%), with MAG15 being dominant especially under optimal digestion conditions (Fig. 1A). Interestingly, nearly half (49.2%) of the binned CAZyme-coding genes were assigned to *Bacteroidetes* MAGs. On average, they accounted for 8.8% ± 1.8% of their genome content, having higher frequency than the MAGs of *Firmicutes* (3.6% ± 1.1%) or *Spirochaetes* (2.9% ± 1.2%). Within *Bacteroidetes* MAGs, 50.1% ± 5.6% of the CAZyme-coding genes were putative GHs, and 15.9% ± 4.0% were putative CEs, showing the high carbohydrate hydrolytic potential of this phylum (Fig. 3A). The distribution into GHs and CEs was quite similar in *Firmicutes* (49.9% ± 12.5% and 12.6% ± 2% of all CAZyme-coding genes) and slightly higher in *Spirochaetes* (59.8% ± 4.7% and 13.9% ± 4.9%). In contrast, reconstructed “*Candidatus* Cloacimonetes” and *Synergistetes* MAGs had higher contents of GTs than GHs (39.7% ± 5.7% versus 21.1% ± 2.75% and 44.3% ± 19.0% versus 31.6% ± 14.7%, respectively). Interestingly, partially reconstructed MAG21, assigned to *Planctomycetes* (65.5% genome completeness) (Table S1), harbored multiple CAZyme-coding genes (representing 8.8% of its total genomic content), mainly assigned to GH127, GH2, GH43, GH5, and CBM35 families.

The levels of diversity of assigned GH families and CBM families were the highest in the case of *Bacteroidetes* (Fig. 3B and C). Around 23.2% of GH families and 24.1% of CBM families were specific to *Bacteroidetes* MAGs only, while roughly 8.7% of GH families and 17.1% of CBM families were absent from *Bacteroidetes* MAGs and present in the other MAGs. Even though the contents of GHs (as well as EC family assignments) differed between *Bacteroidetes* MAGs (Fig. S2) at the phylum level, by adjusting the abundance of single populations, *Bacteroidetes* retained its hydrolytic potential (unchanged GH profile) during the whole experiment.

*Bacteroidetes* MAGs were highly enriched in *sus*-like genes, binning 94.3% of all *sus*-like conserved domains detected in the whole community metagenome (Fig. 3A). On average, *sus*-like genes accounted for 2.4% ± 1.0% of the total gene content in *Bacteroidetes* MAGs, while they were almost absent from the other MAGs. In total, 49 putative PULs were predicted in *Bacteroidetes* MAGs. However, the final PUL number may be larger since its prediction is sensitive to the degree of the fragmentation of draft genomes (21).

### The diversity of CAZyme-coding genes in MAG15 might explain the metagenomic abundance of this species.

**FIG 4:**
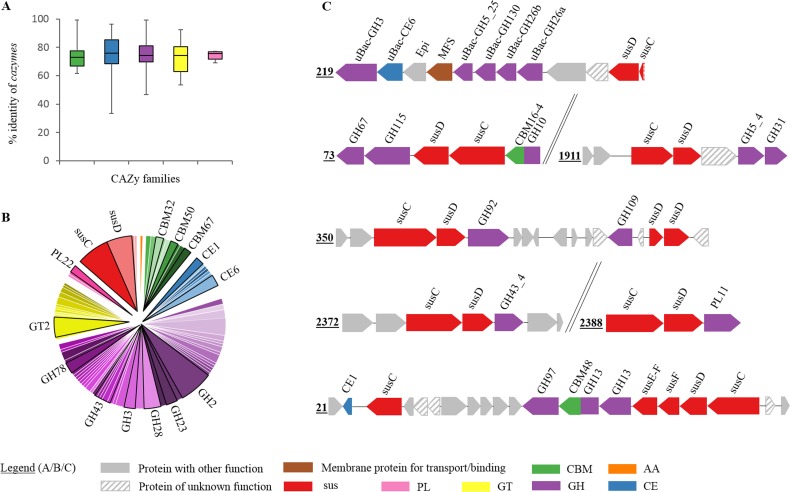
Characterization of the hydrolytic potential of MAG15 assigned to an unknown *Bacteroidetes*. (A) Box plot representations of the percentage identity of the CAZy-coding genes (*cazymes*) from MAG15 to proteins in the NCBI nonredundant protein database. (B) Pie diagram showing the diversity of CAZy-coding genes in MAG15. (C) Putative polysaccharide utilization loci (PULs) identified in the genome of MAG15. Numbers refer to the name of the identified PUL. Epi, *N*-acyl-d-glucosamine 2-epimerase; MFS, major facilitator superfamily. The legend refers to *sus*-like genes, auxiliary enzyme (AA), carbohydrate binding module (CBM), carbohydrate esterase (CE), glycoside hydrolase (GH), glycosyltransferase (GT), and polysaccharide lyase (PL).

MAG15 was quite abundant during the whole anaerobic digestion process and especially under the optimal digestion conditions (Fig. 1A, phase I). Using the database of 8,000 newly reconstructed MAGs from public metagenomic studies (37), we realized that MAG15 was absent from any other environment except our digesters. In addition, the novelty of putative CAZymes from this MAG was confirmed by their low similarities to other CAZyme proteins in the nonredundant protein database (Fig. 4A). CAZyme-coding genes represented 9.1% of the genomic content of MAG15 (Table S1). Among the different CAZy domains identified, GH domains were the most dominant (representing 62.4% of the total CAZy domains in MAG15 genome). CBM and CE domains represented 11.4% and 6.7% of the CAZy domain content, respectively. The GH diversity of MAG15 was high and mainly represented by GH2, GH43, GH28, GH78, and GH3 families (Fig. 4B), possibly allowing the species to cope with the different components of the sugar beet pulp. CBM67, CBM32, and CE6 domain-coding genes were also abundant in the MAG15 genome. Functionally assigned CAZyme-coding genes were represented by 31 EC classes with carbohydrate hydrolytic activity (Fig. S2D). Around 25 *sus*-like coding genes were identified in the genome of this new *Bacteroidetes* sp. and 76% of these genes were localized within PULs (Fig. 4C). In total, seven PULs were identified in MAG15. However, due to the novelty of CAZyme-coding genes, only a few could have been assigned to an EC class, therefore preventing the *in silico* substrate specificity prediction of the identified PULs. PUL219 was the most complete and contained diverse CAZyme-coding genes with putative functions predicted *in silico* to target acetylated glucomannan; therefore, it was further biochemically characterized.

### Biochemical assays confirm predicted CAZy activities of six heterologously expressed proteins present in PUL219.

PUL219 is composed of a putative pair of *susC* and *susD* homologs, one putative protein from the major facilitator superfamily (MFS), and six putative CAZymes belonging to five different families as well as a gene coding for a *N*-acyl-d-glucosamine 2-epimerase (Fig. 4C). Following dbCAN annotation, uBac-GH26a and -b were assigned to the GH26 family, while uBac-GH130, uBac-GH5, uBac-CE6, and uBac-GH3 were assigned to the GH130, GH5, CE6, and GH3 families, respectively. Following the Hotpep analysis, uBac-GH26a and -b were functionally assigned to EC 3.2.1.78 (β-mannanase), uBac-GH130 was assigned to EC 2.4.1.281 (4-*O*-beta-d-mannosyl-d-glucose phosphorylase), uBac-GH5 was assigned to EC 3.2.1.4 (endoglucanase), and uBac-GH3 was assigned to EC 3.2.1.37 (exo-1,4-β-xylosidase). These genes were successfully cloned, and the corresponding proteins were expressed in Escherichia coli Rosetta(DE3) cells.

**FIG 5:**
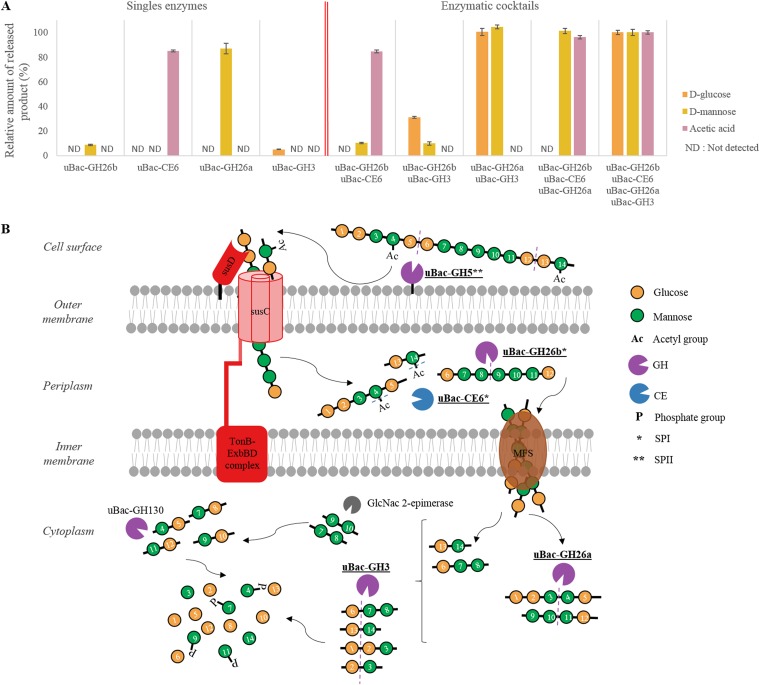
Characterization of PUL219 toward acetylated glucomannan degradation. (A) Release of d-glucose, d-mannose, and acetic acid after enzymatic hydrolysis of acetylated konjac glucomannan. Single enzymes as well as enzymatic cocktails were tested; for all the reactions, enzymatic hydrolysis of substrate lasted 1 h at 37°C. An enzymatic cocktail containing uBac-GH3, uBac-CE6, uBac-GH26a, and uBac-GH26b was taken as a reference to normalize results to 100%. (B) Proposed mechanism of action of the putative acetylated glucomannan-targeting polysaccharide utilization locus PUL219, isolated from a new *Bacteroidetes* species. GlcNac 2-epimerase, *N*-acyl-d-glucosamine 2-epimerase; SPI, signal peptidase I predicted by LipoP, version 1.0; SPII, signal peptidase II predicted by LipoP, version 1.0. Enzymes biochemically characterized in this study are highlighted in bold and underlined.

Results of the enzymatic activity assays and *in silico* signal peptide prediction are summarized in Table S2 and Fig. 5A. As correctly predicted, uBac-GH26a and uBac-GH26b showed capability of endo-action on galactomannan and glucomannan (Table S2; Fig. 5A). They did not show any activity against the tested 4-nitrophenyl derivatives of monosaccharides, including 4-nitrophenyl α-d-mannopyranoside and 4-nitrophenyl β-d-mannopyranoside, showing no mannosidase activity with the tested substrates. Protein uBac-GH5 was active against 4-nitrophenyl β-d-cellobioside and was able to hydrolyze carboxymethyl cellulose (CMC). Moreover, it did not show any activity against 4-nitrophenyl β-d-glucopyranoside, confirming its predicted endoglucanase activity (EC 3.2.1.4). Finally, uBac-CE6 and uBac-GH3 were able to hydrolyze 4-nitrophenyl acetate and 4-nitrophenyl β-d-glucopyranoside, showing, respectively, esterase and β-glucosidase activities. The uBac-GH3 was shown to be a β-glucosidase (EC 3.2.1.21) rather than an exo-1,4-β-xylosidase (EC 3.2.1.37), as initially predicted by the Hotpep analysis. No activity was detected for uBAC-GH130 under the tested conditions and for the tested substrates. However, most of the characterized proteins assigned to this GH family exhibited phosphorylase activity (38). Additionally, the release of d-glucose, d-mannose, and acetic acid from acetylated konjac glucomannan was further assessed by the use of the different enzymatic cocktails and/or cascade reactions (Fig. 5A and Fig. S3). As expected, acetic acid was released when the substrate was treated with esterase (uBac-CE6); however, initial deacetylation did not increase subsequent release of d-glucose and d-mannose compared to the level of nondeacetylated konjac glucomannan. A larger amount of released d-glucose was measured when the enzymatic cocktail contained β-mannanase (cytoplasmic uBac-GH26a) and β-glucosidase (uBac-GH3). For a comparison, a smaller amount of released d-glucose residues was detected when the putatively periplasmic β-mannanase (uBac-GH26b) was combined in a cocktail with β-glucosidase. This finding suggests differential specificity of the two β-mannanases and indicates the formation of, respectively, longer and shorter mannooligosaccharides when the periplasmic and cytoplasmic isoforms are used, respectively. Similarly, a larger amount of released d-mannose was observed when the enzymatic cocktail contained the cytoplasmic form of the β-mannanase combined with the β-glucosidase (uBac-GH3) and independently of the prior substrate deacetylation (Fig. S3). Additionally, using uBac-GH26a as a single enzyme resulted in mannose released from the treated konjac glucomannan. This result in combination with preliminary activity tests with synthetic substrates (e.g., no mannosidase activity detected using 4-nitrophenyl β-d-mannopyranoside) suggests that small-chain oligosaccharides from treated konjac glucomannan could be hydrolyzed by uBac-GH26a, releasing mannoses and shortened manno-oligosaccharides.

## DISCUSSION

### Functional redundancy of AD microbiome.

On one hand, hydrolysis is recognized as a bottleneck of the AD process, especially in the case of highly lignified substrates (8). On the other hand, an increased OLR of easily digestible substrates promotes fast hydrolysis, leading to VFA accumulation and the resulting process failure, known as acidosis. In a previous study, we showed that acidosis, promoted by increasing OLR of dry sugar beet pulp, affected the microbial community structure, causing decreased methane production (Fig. 1A to D) (4). During the acidosis phase, some CO_2_ was still produced at a low rate compared to that under optimal conditions (linked to a lower total number of CAZyme-coding genes observed during acidosis in our study) (Fig. 1), confirming the activity of hydrolytic bacteria at lower pH (39). Following the reestablishment of the optimal AD conditions in the reactor, increased hydrolysis was detected (measured by increased amount of the produced biogas) (Fig. 1), suggesting that the hydrolytic readiness of the microbial community was maintained during the process failure. In addition, we intended to investigate the microbiome functional redundancy with a special focus on its carbohydrate hydrolytic capacity, and to that purpose we applied metagenomics to seven samples taken at different stages of the experiment. Metagenomic results confirmed a significant variation in the abundances of individual bacteria over the experiment, based on the newly reconstructed MAGs (Fig. 1). This is consistent with the previously performed 16S rRNA gene amplicon high-throughput characterization of the microbial community (4). Functional redundancy, known to be widespread in different microbial environments (7), was previously shown at the metaproteome level for a steady-state anaerobic reactor (40). In our study, we showed that despite the differential genomic content of individual MAGs, a strong community functional redundancy was retained even during severe acidosis. This could explain why the reactor was able to quickly recover from acidosis and to promptly restart the production of biogas (functional profiles of genes involved in acetogenic and methanogenic pathways were largely retained as well) (see Fig. S1B and C in the supplemental material). Taxonomic assignment of CAZyme-coding genes showed a shift in the microbial population capable to hydrolyze the substrate along the experiment (Fig. 1B and C). Nevertheless, the genetic potential highlighted at the community-wide level by nearly identical CAZyme-coding gene profiles remained unchanged. This observation somehow fits the recently proposed premise in the context of the human gut of “function first, taxa second” (41). At the same time at the gene expression level, higher variability and sensitivity to perturbation have previously been observed than indicated by the content of the metagenome. Therefore, further metatranscriptomics study would complement the proposed functional redundancy of the AD microbiome by assessing its functional plasticity and, thus, its ability to adapt to perturbations by modulating the expression of the different genes.

### Diversity of CAZymes.

CAZymes are very important to the success of microbes in AD reactors fed with vegetal substrate as they are involved in the digestion of complex polysaccharides, which are particularly abundant in this environment (17). Here, we showed the usefulness of the complementary Hotpep analysis to the dbCAN-mediated CAZyme-coding gene discovery and classification. Indeed, within the abundant GH families (e.g., GH2, GH43, and GH3) (Fig. 1E) different hydrolytic activities were detected on the basis of the assigned EC classes, e.g., α-l-arabinofuranosidase (EC 3.2.1.55), exo-1,4-β-xylosidase (EC 3.2.1.37), endo-1,4-beta-xylanase (EC 3.2.1.8), β-glucosidase (EC 3.2.1.21), and β-galactosidase (EC 3.2.1.23) (Fig. 2C). Still, the biochemical characterization of each new enzyme was necessary to provide correct insights into its saccharolytic capacity and substrate specificity. Sugar beet pulp is composed of diverse carbohydrates, including pectins enriched in arabinans and galactans, cellulose, and hemicelluloses, which comprise small amounts of glucomannans and xyloglucans (42). Accordingly, CAZymes assigned to the most abundant EC categories were predicted to be mainly involved in the hydrolysis of hemicellulosic material and especially (arabino)xylan, (arabino)galactan, and xyloglucan-enriched hemicelluloses. Thus, the ability of the microbiome to hydrolyze sugar beet pulp could be further linked to the abundance of putative β-galactosidases (EC 3.2.1.23) as well as α-l-arabinofuranosidases (EC 3.2.1.55), which were shown to be largely involved in galactan and arabinan degradation (43, 44). Sugar beet pulp constituent-targeting CBMs were also abundant, including pectin-targeting CBM32 or CBM67 that were shown to bind to polygalacturonic acid or α-l-rhamnoside, respectively (45, 46).

The capacity of the microbiome to use different carbon sources was also confirmed by the presence of other CBMs, with CBM50 and CBM56 targeting chitin and peptidoglycan (e.g., fungal and bacterial cell wall components, respectively) being the most abundant. Moreover, family GH109 has often been recorded in metagenomic studies of anaerobic digesters (47), including in our reactor. This family is known to express the α-*N*-acetylgalactosaminidase activity (EC 3.2.1.49), possibly targeting acetylgalactosamine, which is a component of bacterial cell walls (37). On one hand, the abundance of genes assigned to this family, together with the presence of CBM50 and CBM56 domains, might be linked to the expected high turnover rates of bacterial biomass resulting from high competition in this environment (48). On the other hand, only a few GH109-classified CAZyme-coding genes were assigned to the respective EC 3.2.1.49 category (0.4% ± 0.3% of metagenomic abundance) (Fig. 2C). Therefore, further investigation is required to confirm the specific (hydrolytic) activity of this family of proteins. The presence of AA coding genes, including AA2-containing class II lignin-modifying peroxidases, indicates the potential to decompose lignin by some anaerobic digester microbes. Demethylation of lignin was suggested for some *Spirochaetes* isolated from the termite gut (49), which is in accordance to our *Spirochaetes* MAGs being enriched in AA coding genes.

### *Bacteroidetes* and their PULs.

Similar to findings of previous studies of AD microbiomes (10, 50, 51), *Bacteroidetes* MAGs were shown to be among of the most dominant in the reconstructed metagenome representing the digester microbial community. Indeed, higher genomic content and functional diversity of hydrolytic genes (GHs and ECs) in *Bacteroidetes* MAGs (Fig. 3A and Fig. S4) could ensure digestibility of a wide range of substrates, thus favoring their abundance in AD reactors under certain environmental conditions (10). Individual *Bacteroidetes* MAGs were characterized with distinct CAZyme-coding gene functional profiles suggesting niche differentiation (different nutritional requirements) of the different species (Fig. S2). However, at the phylum level the *Bacteroidetes* population, by regulating the abundance of single individuals, was capable of ensuring the stability of gene functional profiles, thus largely contributing to the community-wide functional redundancy. *Bacteroidetes* and some other bacteria (e.g., *Firmicutes* and representatives of *Spirochaetes*, *Lentisphaerae*, *Planctomycetes*, and *Thermotogae*) that harbor multiple CAZyme-coding genes in their genomes (Table S1) seem responsible for complex carbohydrate degradation (14). Interestingly, *Planctomycetes* MAG21 harbors a CAZy profile similar to that of *Bacteroidetes* MAGs with a relatively high CAZyme-coding gene content (Fig. 3A). Even though the role of this bacterium in anaerobic digestion has not been widely discussed, previous studies suggest the involvement of *Planctomycetes* in degradation of chitin and cellulose in agricultural soil (52) and humus decomposition in the termite gut (53). These observations might indicate a similar hydrolytic potential to *Bacteroidetes* and the importance of *Planctomycetes* in the anaerobic digestion process (due to its ability to decompose recalcitrant organic matter). In contrast, “*Ca*. Cloacimonetes” and *Synergistetes* have different CAZy profiles, mainly enriched in GTs, and seem of less importance for carbohydrate hydrolysis. Nevertheless, secondary ion mass spectrometry-*in situ* hybridization using an iodine-labeled oligonucleotide probe combined with high-resolution nanometer-scale secondary-ion mass spectrometry (SIMS) supported the role of “*Ca*. Cloacimonetes” in the fermentative digestion of cellulose (54). Owing to its status of a candidate phylum and to the recently proposed diversity of “*Ca*. Cloacimonetes” in the different full-scale AD reactors (10), a broad range of functional diversity may be expected within this phylum.

MAG15 assigned to an unknown *Bacteroidales* was characterized with relatively high community abundance in the reactor during the whole AD experiment (Fig. 1A). According to its CAZy profile (Fig. 4B and C), it showed potential to digest a broad range of polysaccharides present in sugar beet pulp. CAZyme-coding genes assigned to the family GH78 known to have α-l rhamnosidase activity (EC 3.2.1.40) acting on rhamnogalacturonan, a component of pectins (44), were present in multiple copies in its genome. In addition, the presence of numerous CAZyme-coding gene modules from the CBM67 family having α-l-rhamnose binding activity (46) confirms the potential of this bacterium to target pectins (Fig. 4B). The presence of (hetero)xylan targeting CAZyme genes, including the putative enzymes attacking the backbone (endoxylanases and xylosidases assigned to families GH2, -3, -10, and -43, e.g.), as well as arabinases (e.g., GH2 and 43) and galactosidases (e.g., GH2), confirms the ability of MAG15 to decompose different forms of xylans. Many novel *Bacteroidetes* were shown to contain various sets of PULs predicted to be involved in complex hydrolysis of diverse carbohydrates (19), changing our understanding of polysaccharide metabolism inside this phylum. The partially reconstructed MAG15 contains multiple PULs, including PUL73 predicted to target xylan and starch-targeting PUL21. PUL219, containing the highest diversity of putative CAZyme-coding genes, was additionally biochemically validated to target (acetylated) glucomannan. Glucomannan is a polysaccharide composed of β-D-1,4-linked mannose and glucose monomers (possible combinations include glucose-mannose, glucose-glucose, mannose-glucose, and mannose-mannose linkages), with side chain acetyl groups (Fig. 5B) (55, 56). Previously biochemically validated PULs included a galactomannan-targeting PUL from a marine *Bacteroidetes* (57) and a cellulose-degrading one from a rumen-isolated bacterium (58). Here, we propose a model of PUL-assisted acetylated glucomannan degradation for a *Bacteroidetes* species (Fig. 5B). This model was reconstructed based on the activity tests performed in this study using purified proteins and complementary *in silico* predictions, as described below. According to the predicted presence of a lipoprotein signal peptidase II and a cleavage site within uBac-GH5 sequence, this endoglucanase is most probably attached to the outer membrane, extracellularly digesting acetylated glucomannans and releasing acetylated glucomannan oligosaccharides (59, 60). As *susD* is attached to the outer membrane, it binds the resulting substrate and directs it to the *susC* transporter, which passes it to the periplasm (61). A signal peptidase I and a cleavage site were *in silico* predicted in both uBac-GH26b and uBac-CE6, suggesting their migration from the cytoplasm to the periplasm (62). In the periplasm, β-mannanase (uBac-GH26b) further hydrolyzes the acetylated glucomannan oligomers while acetyl groups are removed by the action of an esterase (uBac-CE6). Even shorter oligosaccharides may now be transported through the inner membrane with the help of the MFS (63). In the cytoplasm, short chains of glucomannan are further digested by uBac-GH26a (second β-mannanase), possibly releasing mono-, di-, and trisaccharides (64). These oligosaccharides are further hydrolyzed by uBac-GH3 (β-glucosidase), releasing glucose, mannose, and mannobiose. *N*-Acyl-d-glucosamine 2-epimerase transforms mannobiose into β-d-mannosyl-(1→4)-d-glucose (MannosylGlu). Hydrolysis of MannosylGlu to glucose and mannose-1-phosphate is mediated by uBac-GH130 (presumed to be the 4-*O*-beta-d-mannosyl-d-glucose phosphorylase) (65, 66). A similar galactomannan-targeting PUL from Bacteroides ovatus has been previously characterized (67), including two β-mannanases from the GH26 family (BoMan26A and BoMan26B). In that study, BoMan26B was shown to release longer-chain oligosaccharides than BoMan26A, similarly to uBac-GH26b and uBac-GH26a as proposed in our model (Fig. 5). Phylogenetic analysis of several mannanases (including previously characterized BoMan26A and BoMan26B) confirmed the presence of two distinct mannanase clusters inside the GH26 family, showing the differential clustering of uBac-GH26a and uBac-GH26b, which are closely related to BoMan26A and BoMan26B, respectively (Fig. S5).

Further characterization of novel PULs should bring even more interesting insights into the complex carbohydrate metabolism in *Bacteroidetes*. Specific substrate targeting ready-to-use enzymatic cocktails naturally selected in PULs might be of high interest to the biotechnology sector as well, especially for developing biomass-based green chemistry. For example, acetylated glucomannan is the main hemicellulosic component of the secondary cell walls in soft woods (68), which are common substrates for pulp and paper production. A nature-evolved cocktail of acetylated glucomannan-targeting enzymes, as characterized in, e.g., PUL219, might be an interesting alternative to the currently used chemical pretreatments applied in these industries.

## MATERIALS AND METHODS

### Sampling, metagenomics, and data processing.

Samples were taken from an R3 lab-scale continuously stirred tank reactor (CSTR) of 100-liter capacity fed with dry sugar beet pulp as described in a previous study. Decreasing HRT and increasing OLR resulted in a pH decrease and lower biomass-to-methane conversion ratio (4). In total, seven time points were analyzed, corresponding to the different stages of the experiment (Fig. 1). Total genomic DNA was extracted with a PowerSoil DNA isolation kit (MoBio Laboratories, Inc.), according to the manufacturer’s instructions. Metagenomic DNA libraries were constructed, sequenced, and analyzed as previously described (31). Binning of assembled contigs resulted in over 30 MAGs with average genome completeness of 76.6% ±19.8% and contamination below 5%. MAGs described in this study correspond to the bacterial bins listed in Table 2 in a previous publication (31). The taxonomic assignment of MAGs was done with Phylophlan (69). Prodigal was used to predict coding sequences on the coassembled contigs (70). CAZyme-coding genes were searched with the dbCAN (dbCAN-fam-HMMs.txt.v6 [27]) against a CAZy database (http://www.cazy.org/). The resulting CAZyme-coding genes were manually curated. A difference in abundances of MAGs/GHs/ECs was tested by a chi-square test. PULs were predicted according to the PUL database (71), and the presence of at least one *susD* or *susC* gene was mandatory for partial PUL prediction. Homology to peptide pattern (Hotpep) was used to assign an EC class to the identified CAZymes (72). Taxonomic and functional annotation of the coassembled contigs was done with IMG-MER (73) and corresponds to the project identification number 3300002079. The most complete MAGs were reannotated with RAST (79).

### CAZyme-coding gene heterologous expression in E. coli.

Details for gene selection, isolation, and cloning are shown in Text S1 in the supplemental material. For CAZyme-coding gene expression, plasmid pET-52b(+) (Millipore Corporation, Billerica, MA, USA) and the E. coli Rosetta(DE3) strain (Millipore Corporation, Billerica, MA, USA) were used. Genes were ligated into pET-52b(+) vector and introduced in E. coli by heat shock. An isolated colony (the insert was determined by sequencing) was grown in LB medium containing ampicillin (LB-Amp) overnight at 37°C with 250 rpm shaking. In total, 100 μl of this culture was transferred into a fresh 100 ml of LB-Amp. Cells were grown at 37°C with shaking at 250 rpm to an optical density at 600 nm (OD_600_) of 0.5, and isopropyl-β-d-thiogalactopyranoside (IPTG; ThermoFisher, Waltham, MA, USA) was added at final concentration of 0.5 mM to induce the expression of recombinant proteins. The culture was maintained for 20 h at room temperature and 250 rpm.

Cells were collected by centrifugation at 5,000 × *g* at 4°C for 15 min. Supernatant was stored at −20°C, and the cell pellet was redissolved in an appropriate lysis buffer (50 mM NaH_2_PO_4_, 300 mM NaCl, 10 mM imidazole, pH 8). As a negative control, an empty pET-52b(+) vector was cloned, expressed, and processed as a recombinant protein sample.

Using a method adapted from previous studies (74, 75), cell lysis was achieved using a Sonicator VC750 (Sonics & Materials, Inc., Newtown, CT, USA) with a 2-min pulse (1 s on/1s off) followed by a 2-min pause (40% of amplitude). This step of sonication was repeated twice. Samples were transferred into 2-ml tubes and centrifuged for 15 min at 16,000 × *g* at 4°C. The liquid part was filtered using an Acrodisc 13-mm syringe filter with a 0.2-μm-pore-size Supor membrane (Millipore Corporation, Billerica, MA, USA). Protein detection and partial purification were performed using a histidine tag located at the C terminus. Initial detection on cell lysates was performed using Western blotting. Separation was done using 10% Mini-Protean TGX precast protein gels (Bio-Rad, Hercules, CA, USA). Transfer was performed with a Trans-Blot Turbo transfer system (Bio-Rad, Hercules, CA, USA). Detection was carried out using an appropriate antibody labeled with horseradish peroxidase (HRP) (6×His tag polyclonal antibody) (ThermoFisher, Waltham, MA, USA). Ni-nitrilotriacetic acid (NTA) agarose matrix was used to load polypropylene columns for partial purification according to the supplier’s instructions (Qiagen, Hilden, Germany). Protein quantification was determined using a reducing agent and detergent-compatible (RC DC) protein assay (Bio-Rad, Hercules, USA) and bovine serum albumin as a standard. Proteins were identified, and details can be found in Text S2 in the supplemental material.

### Signal peptide predication and activity assays.

Signal peptides were predicted using LipoP, version 1.0 (http://www.cbs.dtu.dk/services/LipoP/) (76) Enzymatic assays were performed using 4-nitrophenyl derivatives as substrates (Sigma-Aldrich, Saint Louis, MO, USA). Initially, 50 μl of partially purified protein solution was incubated with 100 μl of citrate phosphate buffer, pH 7 (0.1 M citric acid, 0.2 M dibasic sodium phosphate), and 50 μl of substrate (Table S3). A microplate was incubated at 37°C in a Tecan Spark 20 M microplate reader (Tecan, Mannedorf, Switzerland). Th rate of released 4-nitrophenol was monitored at 405 nm. Assays were performed in triplicate.

Following previous studies (77, 78), enzymatic activity was also assessed by the release of reducing sugars. CMC (Sigma-Aldrich, Saint Louis, USA), arabinoxylan, galactomannan, glucomannan, and xylan (Megazyme, Wicklow, Ireland) were used as substrates. Briefly, 100 μl of partially purified enzyme solution was incubated with 50 μl of citrate phosphate buffer, pH 7 (0.1 M citric acid, 0.2 M dibasic sodium phosphate), and 100 μl of substrate at 37°C for 30 min (Table S3). The concentrations of reducing sugars were determined applying the Somogyi-Nelson method, and absorbance was read at 620 nm using Specord Plus spectrophotometer (Analytik Jena, Jena, Germany). Assays were performed in triplicates.

Single enzymes as well as enzymatic cocktails were used for the pretreatment of glucomannan to determine the release of d-glucose, d-mannose, and acetic acid. Briefly, pretreatment was carried out in 500 μl as follows: 250 μl of substrate, 50 μl of buffer, and 50 μl of each enzyme assessed; final volume was reached by addition of water if necessary. The reaction mixture was incubated at 37°C for 1 h. If a cascade reaction was tested, a second enzyme (enzymatic set) was added after 1 h of pretreatment and reincubated for 1 h at 37°C. The release of d-glucose, d-mannose, and acetic acid was determined using commercially available kits (Megazyme, Wicklow, Ireland), according to the supplier’s instructions. An enzymatic cocktail containing uBac-GH3, uBac-CE6, uBac-GH26a, and uBac-GH26b was taken as a reference to normalize results to 100%.

### Data availability.

The most complete MAGs can be accessed using a RAST (http://rast.nmpdr.org) guest account under the following identification numbers: 6666666.364478 (MAG1), 6666666.364479 (MAG2), 6666666.364480 (MAG3), 6666666.364481 (MAG4), 6666666.364482 (MAG5), 6666666.364483 (MAG6), 6666666.364484 (MAG7), 6666666.364485 (MAG8), 6666666.364486 (MAG9), 6666666.364487 (MAG10), 6666666.364489 (MAG11), 6666666.364490 (MAG12), 6666666.364491 (MAG13), 6666666.364493 (MAG14), 6666666.364494 (MAG15), 6666666.364495 (MAG16), 6666666.364496 (MAG17), 6666666.364497 (MAG18), 6666666.364498 (MAG19), 6666666.364499 (MAG20), 6666666.364500 (MAG21), 6666666.364502 (MAG23), 6666666.364503 (MAG25), 6666666.364504 (MAG30), and 6666666.364505 (MAG31).

## Supplementary Material

Supplemental file 1
