# Supplementary material for: Carbohydrate Hydrolytic Potential and Redundancy of an Anaerobic Digestion Microbiome Exposed to Acidosis, as Uncovered by Metagenomics
Source: Appl Environ Microbiol. 2019 Jul 18;85(15):e00895-19. doi: 10.1128/AEM.00895-19 (PMC6643232; doi:10.1128/AEM.00895-19)
Supplement: Supplemental file 1 [file AEM.00895-19-s0001.pdf]

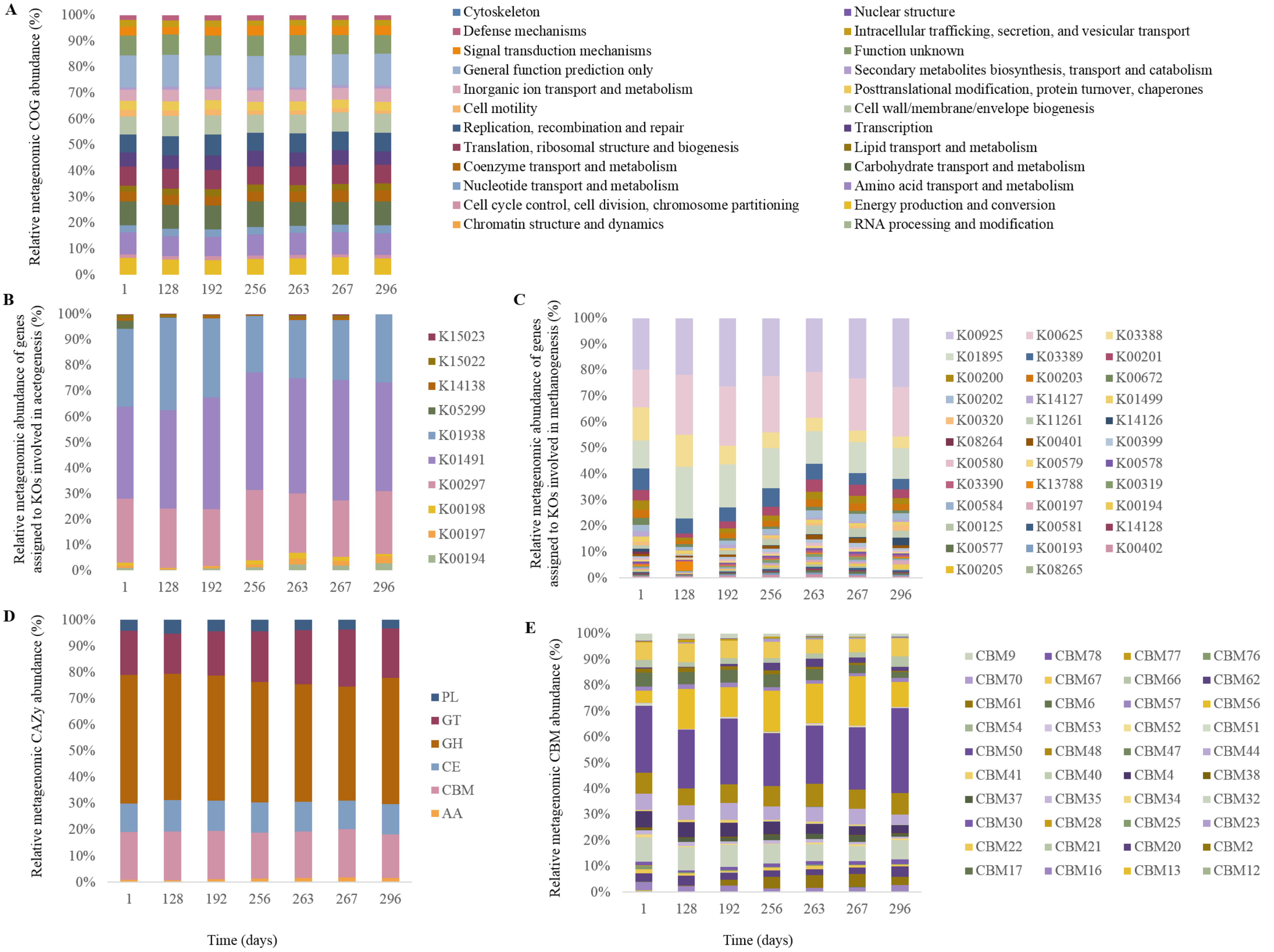
A
B
C
D
E
100%
90%
80%
70%
60%
50%
40%
30%
20%
10%
0%
1
128
192
256
263
267
296
Relative metagenomic COG abundance (%)
Cytoskeleton
Nuclear structure
Defense mechanisms
Intracellular trafficking, secretion, and vesicular transport
Signal transduction mechanisms
Function unknown
General function prediction only
Secondary metabolites biosynthesis, transport and catabolism
Inorganic ion transport and metabolism
Posttranslational modification, protein turnover, chaperones
Cell motility
Cell wall/membrane/envelope biogenesis
Replication, recombination and repair
Transcription
Translation, ribosomal structure and biogenesis
Lipid transport and metabolism
Coenzyme transport and metabolism
Carbohydrate transport and metabolism
Nucleotide transport and metabolism
Amino acid transport and metabolism
Cell cycle control, cell division, chromosome partitioning
Energy production and conversion
Chromatin structure and dynamics
RNA processing and modification
Relative metagenomic abundance of genes assigned to KOs involved in acetogenesis (%)
K15023
K15022
K14138
K05299
K01938
K01491
K00297
K00198
K00197
K00194
Relative metagenomic abundance of genes assigned to KOs involved in methanogenesis (%)
K00925
K00625
K03388
K01895
K03389
K00201
K00200
K00203
K00672
K00202
K14127
K01499
K00320
K11261
K14126
K08264
K00401
K00399
K00580
K00579
K00578
K03390
K13788
K00319
K00584
K00197
K00194
K00125
K00581
K14128
K00577
K00193
K00402
K00205
K08265
Relative metagenomic CAZy abundance (%)
PL
GT
GH
CE
CBM
AA
Time (days)
Relative metagenomic CBM abundance (%)
CBM9
CBM78
CBM77
CBM76
CBM70
CBM67
CBM66
CBM62
CBM61
CBM6
CBM57
CBM56
CBM54
CBM53
CBM52
CBM51
CBM50
CBM48
CBM47
CBM44
CBM41
CBM40
CBM4
CBM38
CBM37
CBM35
CBM34
CBM32
CBM30
CBM28
CBM25
CBM23
CBM22
CBM21
CBM20
CBM2
CBM17
CBM16
CBM13
CBM12
Time (days)

Fig. S1: Metagenomics-assisted characterisation of the functional community potential throught the anaerobic digestion (AD) experiment.

(A) Relative metagenomic abundance of the different clusters of orthologous groups (COG) coloured according to the COG group. (B) Relative metagenomic abundance of the different Kyoto Encyclopedia of Genes and Genomes (KEGG) orthology (KO) involved in the acetogenesis pathway coloured according to the KO number. (C) Relative metagenomics abundance of the different KEGGs involved in the methanogenesis (including acetoclastic and hydrogenotrophic pathways) coloured according to the KO number. (D) Relative metagenomic abundance of the different CAZy families coloured according to the CAZy family. The differents CAZy families represented are; auxiliary enzyme (AA); Carbohydrate binding module (CBM); Carbohydrate esterase (CE); Glycoside hydrolase (GH); Glycosyltransferase (GT); Polysaccharide lyase (PL). (E) Relative metagenomic abundance of the different CBM families coloured according to the CBM family.

Note: Small variability of the functional profile of genes involved in methanogenesis was related to the overall decrease of genes encoding for the heterodisulfide reductase (hydrogenotrophic pathway) during acidosis.

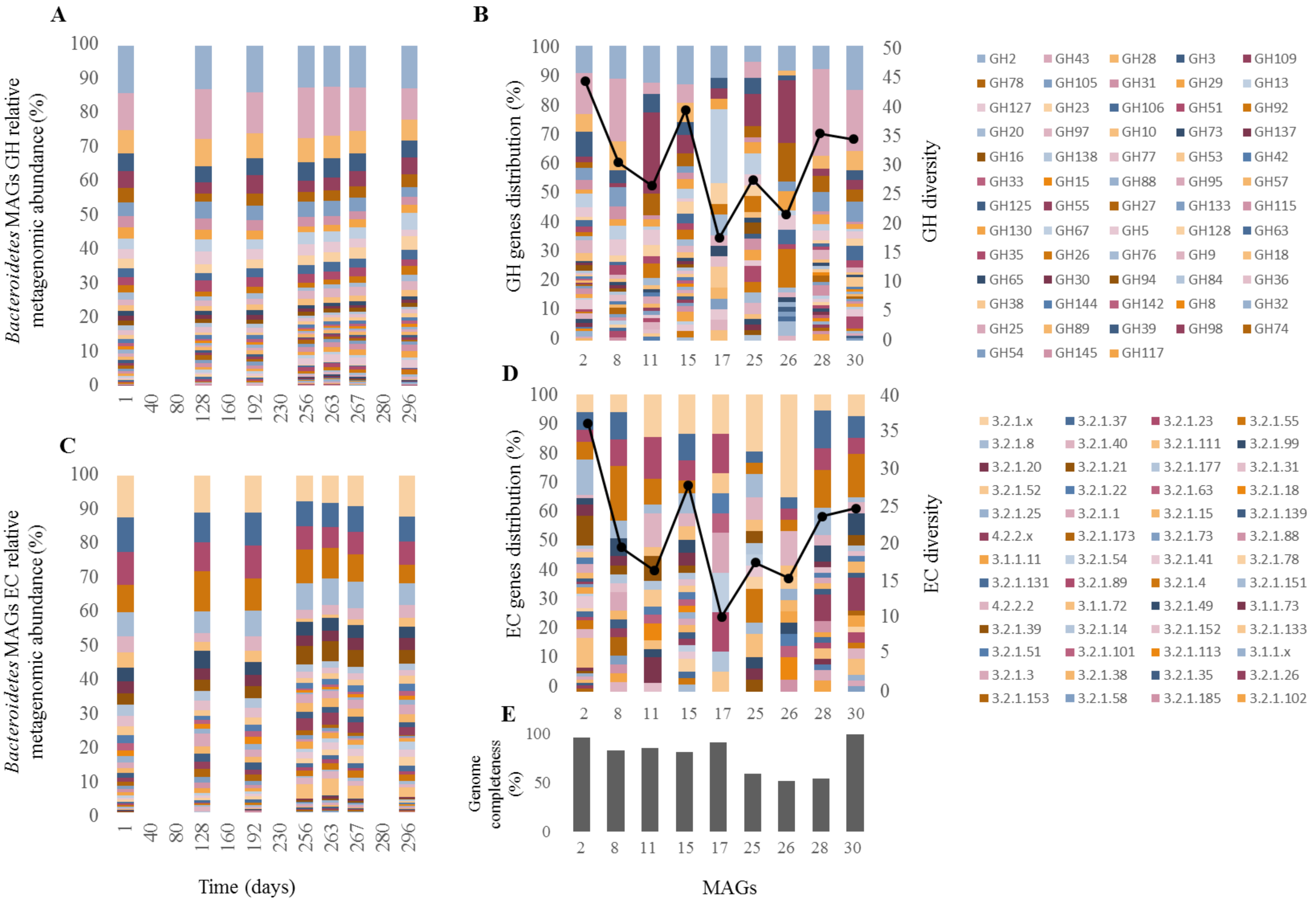
A
B
C
D
E
Bacteroidetes MAGs GH relative metagenomic abundance (%)
Bacteroidetes MAGs EC relative metagenomic abundance (%)
Time (days)
1 40 80 128 160 192 230 256 263 267 280 296
GH genes distribution (%)
GH diversity
EC genes distribution (%)
EC diversity
Genome completeness (%)
MAGs
2 8 11 15 17 25 26 28 30
GH2 GH43 GH28 GH3 GH109
GH78 GH105 GH31 GH29 GH13
GH127 GH23 GH106 GH51 GH92
GH20 GH97 GH10 GH73 GH137
GH16 GH138 GH77 GH53 GH42
GH33 GH15 GH88 GH95 GH57
GH125 GH55 GH27 GH133 GH115
GH130 GH67 GH5 GH128 GH63
GH35 GH26 GH76 GH9 GH18
GH65 GH30 GH94 GH84 GH36
GH38 GH144 GH142 GH8 GH32
GH25 GH89 GH39 GH98 GH74
GH54 GH145 GH117
3.2.1.x 3.2.1.37 3.2.1.23 3.2.1.55
3.2.1.8 3.2.1.40 3.2.1.111 3.2.1.99
3.2.1.20 3.2.1.21 3.2.1.177 3.2.1.31
3.2.1.52 3.2.1.22 3.2.1.63 3.2.1.18
3.2.1.25 3.2.1.1 3.2.1.15 3.2.1.139
4.2.2.x 3.2.1.173 3.2.1.73 3.2.1.88
3.1.1.11 3.2.1.54 3.2.1.41 3.2.1.78
3.2.1.131 3.2.1.89 3.2.1.4 3.2.1.151
4.2.2.2 3.1.1.72 3.2.1.49 3.1.1.73
3.2.1.39 3.2.1.14 3.2.1.152 3.2.1.133
3.2.1.51 3.2.1.101 3.2.1.113 3.1.1.x
3.2.1.3 3.2.1.38 3.2.1.35 3.2.1.26
3.2.1.153 3.2.1.58 3.2.1.185 3.2.1.102

Fig. S2: Metagenomics-assisted characterisation of *Bacteroidetes* MAGs and their carbohydrate hydrolytic potential throughout the anaerobic digestion (AD) experiment. (A) Relative metagenomic abundance of the different GHs coloured according to the assigned GH family and throughout the digestion experiment. (B) Distribution of the annotated GH genes for the different *Bacteroidetes* MAGs, coloured according to the assigned GH family. The number of assigned GH families (i.e. GH diversity) is represented by a black solid line. (C) Relative metagenomic abundance of the different GHs additionally functionally assigned to an EC category and coloured according to the EC category. (D) Distribution of the assigned EC categories among the different *Bacteroidetes* MAGs, coloured according to the EC category and throughout the digestion experiment. The number of EC functional categories (i.e. EC diversity) is represented by a black solid line. (E) Percentage of genome completeness for the different *Bacteroidetes* MAGs.

Remark: Due to incompleteness of some *Bacteroidetes* MAGs, the diversity measurements for the different GH families (respectively EC categories) can be higher than shown on the respective graphs.

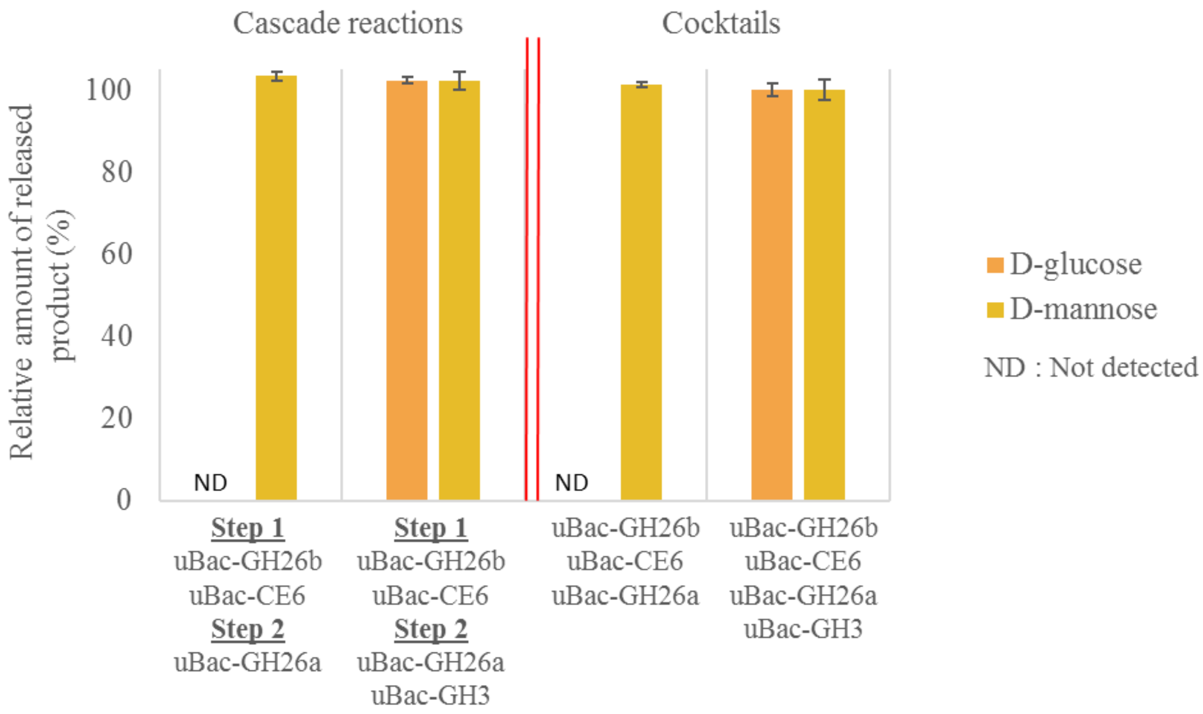

Fig. S3: Release of D-glucose and D-mannose after enzymatic hydrolysis of acetylated konjac glucomannan. Cascade reactions and cocktails were compared. Cascade reaction is defined as follow, first hydrolysis of the substrate (one hour at 37°C) by the enzymes specified in step 1, followed by a second hydrolysis (one hour at 37°C) by the enzyme(s) specified in step 2. Cocktail is defined as follow, enzymes were added and a single incubation step (one hour at 37°C) was performed in order to hydrolyse the substrate.

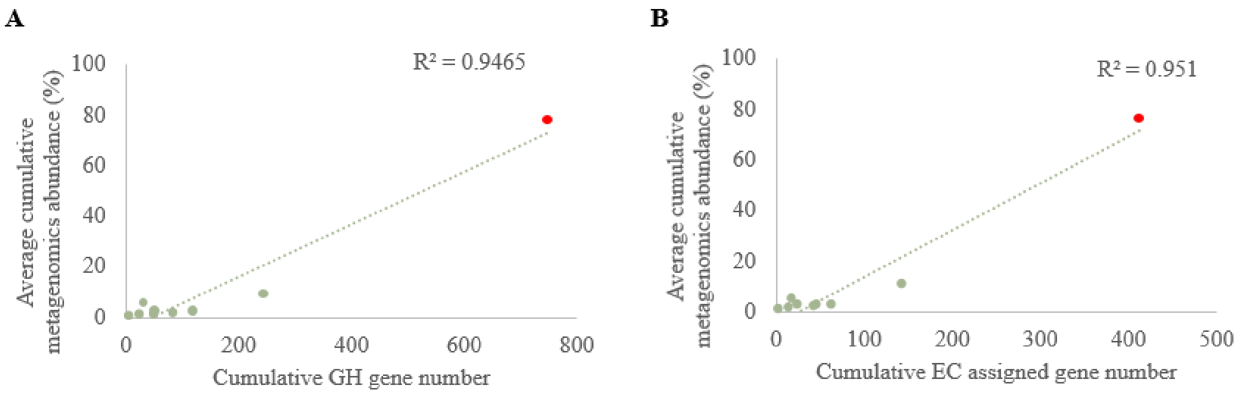

Fig. S4: Correlation between the average cumulative metagenomic abundance of MAGs represented at the phylum level and the cumulative number of their carbohydrate hydrolytic genes, shown at the GH family level (A) and further supported by the assigned EC category (B). *Bacteroidetes* phylum is represented by a red dot.

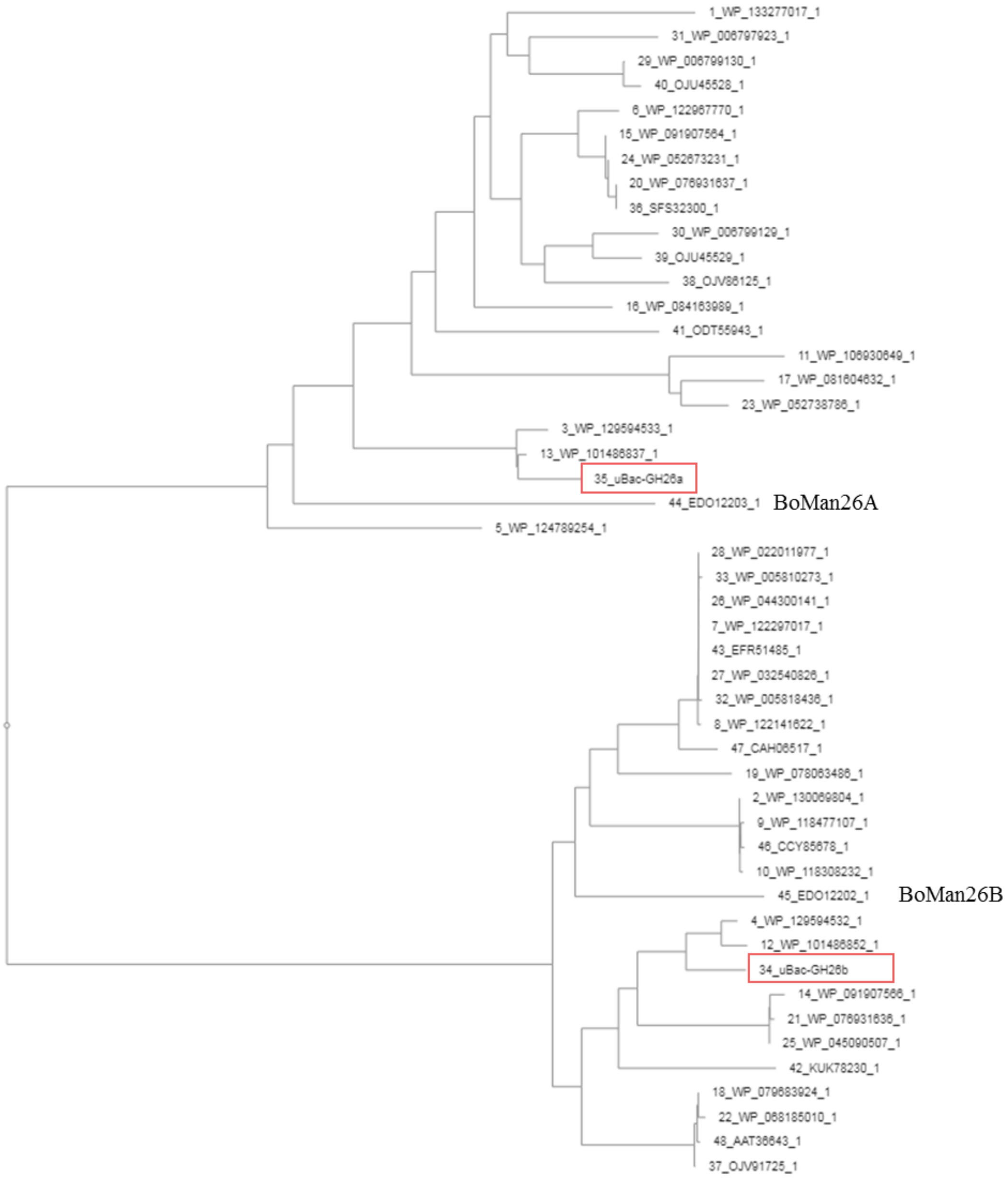

Fig. S5: uBac-GH26a and uBac-GH26b sequence similarities including mannanases from NCBI database. Sequence alignment and phylogenetic tree was generated using the MAFFT server https://mafft.cbrc.jp/alignment/software. BoMan26A and BoMan26B refers to two mannanases isolated from a galactomannan-targeting PUL (1). uBac-GH26a and uBac-GH26b are highlighted in red.

1. Bagenholm V, Reddy SK, Bouraoui H, Morrill J, Kulcinskaja E, Bahr CM, Aurelius O, Rogers T, Xiao Y, Logan DT, Martens EC, Koropatkin NM, Stalbrand H. 2017. Galactomannan catabolism conferred by a polysaccharide utilization locus of

Bacteroides ovatus: Enzyme synergy and crystal structure of a β-mannanase. J Biol Chem 292:229–243

Table S1: Analysis of the different MAGs

Phylophlan	MAG number	Proteins coding genes	sus-like genes	CAZys coding genes	% CAZys in the MAG	% Completeness	% Metagenomic abundance
Bacteria; Bacteroidetes	2	2263	97	222	9.8	96.6	6.3
Bacteria; Bacteroidetes; Bacteroidia; Bacteroidales	8	1515	41	151	10.0	82.9	8.8
Bacteria; Bacteroidetes; Bacteroidia; Bacteroidales; Rikenellaceae; Alistipes	11	1881	29	151	8.0	85.9	1.2
Bacteria; Bacteroidetes; Bacteroidia; Bacteroidales	15	1461	23	135	9.1	81.6	12
Bacteria; Bacteroidetes; Bacteroidia; Bacteroidales; Rikenellaceae; Alistipes	17	1320	25	62	4.7	91.2	1.2
Bacteria; Bacteroidetes; Bacteroidia; Bacteroidales; Rikenellaceae; Alistipes	25	1276	32	104	8.2	59	0.5
Bacteria; Bacteroidetes; Bacteroidia; Bacteroidales	26	1237	34	107	8.7	51.3	0.3
Bacteria; Bacteroidetes; Bacteroidia; Bacteroidales	28	1726	60	160	9.3	54.5	0.6
Bacteria; Bacteroidetes; Bacteroidia; Bacteroidales; Rikenellaceae; Alistipes	30	1914	9	228	11.9	99.5	3.4
Bacteria; Firmicutes; Clostridia; Clostridiales	3	1784	0	73	4.1	95.3	1.6
Bacteria; Firmicutes; Clostridia	4	2099	1	89	4.2	94.9	2.6
Bacteria; Firmicutes; Clostridia	12	2064	0	74	3.6	89.8	2.4
Bacteria; Firmicutes; Clostridia	16	1615	0	74	4.6	63.9	0.7
Bacteria; Firmicutes; Clostridia	20	1307	1	45	3.4	60.4	0.5
Bacteria; Firmicutes; Clostridia; Clostridiales	22	1040	0	58	5.6	62.0	0.3
Bacteria; Firmicutes; Clostridia; Clostridiales	23	1034	0	43	4.2	51.8	0.6
Bacteria; Firmicutes; Clostridia	27	1216	0	27	2.2	49.5	0.3
Bacteria; Cloacimonetes	1	1250	3	40	3.2	98.9	22.3
Bacteria; Cloacimonetes	13	1404	2	62	4.4	87.3	1.1
Bacteria; Cloacimonetes	31	1443	2	60	4.2	95.6	1.7
Bacteria; Spirochaetes; Spirochaetes; Spirochaetales	7	1945	1	90	4.6	66.7	1.1
Bacteria; Spirochaetes; Spirochaetes; Spirochaetales	14	1889	0	62	3.3	83.8	0.8
Bacteria; Spirochaetes; Spirochaetes; Spirochaetales; Treponemaceae; Treponema	18	1621	0	47	2.9	76.3	0.6
Bacteria; Synergistetes; Synergistia; Synergistales; Synergistaceae	5	1790	1	68	3.8	94.8	1.5
Bacteria; Synergistetes; Synergistia; Synergistales; Synergistaceae	9	1708	2	44	2.6	92.8	0.7
Bacteria; Synergistetes; Synergistia; Synergistales; Synergistaceae	10	1368	2	29	2.1	92.2	1
Bacteria; Lentisphaerae; Lentisphaerae	19	1736	0	113	6.5	77.3	1.4
Bacteria; Planctomycetes; Planctomycetacia	21	1935	6	171	8.8	65.5	0.6
Bacteria; Thermotogae; Thermotogae; Thermotogales; Thermotogaceae	24	1273	0	78	6.1	66.8	0.4
Archaea; Euryarchaeota; Methanomicrobia; Methanomicrobiales	6	1447	0	28	1.9	92.2	1.5

Table S2: Results of the enzymatic assays and EC number prediction

		Activity (10^{-3} U/mg ± SD)					
		uBac-GH26a	uBac-GH26b+	uBac-GH130	uBac-GH5++	uBac-CE6+	uBac-GH3
Substrate type	Substrate	EC.3.2.1.78*	EC.3.2.1.78*	EC. 2.4.1.281*	EC. 3.2.1.4*	NA	EC.3.2.1.37*
Polysaccharide	Xylan	-	-	-	-	-	-
	Galactomannan	94.86 ± 6.08	90.68 ± 5.28	-	-	-	-
	Arabinoxylan	-	-	-	-	-	-
	Carboxymethyl cellulose	-	-	-	19.05 ± 0.51	-	-
	Glucomannan	102.84 ± 1.06	31.39 ± 1.52	-	-	-	-
4-Nitrophenyl	α-D-mannopyranoside	-	-	-	-	-	-
	β-D-glucopyranoside	-	-	-	-	-	177.00 ± 7.52
	β-D-xylopyranoside	-	-	-	-	-	-
	β-D-mannopyranoside	-	-	-	-	-	-
	α-L-arabinofuranoside	-	-	-	-	-	-
	β-D-cellobioside	-	-	-	9.94 ± 0.88	-	-
	α-D-galactopyranoside	-	-	-	-	-	-
	acetate	-	-	-	-	30215.70 ± 0.20	-
	α-D-glucopyranoside	-	-	-	-	-	-

Results are expressed in U/mg where one unit is defined as the amount of protein required to release one µmol of substrate per minute.

* EC number prediction based on Hotpep analysis

NA: Not assigned

+ Signal peptidase I and cleavage site predicted using LipoP v1.0 server

++ Lipoprotein signal peptide II and cleavage site predicted using LipoP v1.0 server

Table S3: Concentrations of the substrates used for the enzymatic assays

Substrate	Stock concentration (mM)
4-nitrophenyl α-D-mannopyranoside	20
4-nitrophenyl β-D-glucopyranoside	40
4-nitrophenyl β-D-xylopyranoside	40
4-nitrophenyl β-D-mannopyranoside	20
4-nitrophenyl α-L-arabinofuranoside	20
4-nitrophenyl β-D-cellobioside	10
4-nitrophenyl α-D-galactopyranoside	40
4-nitrophenyl acetate	4
4-nitrophenyl α-D-glucopyranoside	20
**Substrate**	**Stock concentration (g/L)**
CMC	10
Arabinoxylan	12
Galactomannan	8
Glucomannan	8
Xylan	18

Text S1: Genes selection, isolation and cloning

Following the bioinformatics analyses, genes of interest were identified and amplified by polymerase chain reaction (PCR), using a Veriti™ 96 wells Thermal cycler (Applied Biosystems, Foster City, USA). Amplicons were generated using the Q5 High-Fidelity Hot Start (New England Biolabs Inc., Ipswich, USA) and according to the thermal profile: initial denaturation was made at 98°C for 5 seconds, followed by annealing for 30 seconds with temperature depending on the melting temperature of the primers (Tm), and the final extension was carried out at 72°C for 30 seconds. Thirty six cycles of PCR were performed. PCR products were purified using the PCR purification kit (Qiagen, Hilden, Germany) and concentrations were determined by NanoDrop 1000 Spectrometer (Thermofisher, Waltham, USA).

Genes were then cloned into pGem-t-easy vector using the respective kit (Promega, Madison, USA) followed by transformation of JM109 High Efficiency Competent Cells, allowing blue/white screening of colonies. Positive colonies were confirmed by a colony PCR and the respective plasmids from over-night cultures were purified using GeneJET Plasmid Miniprep Kit (Thermofisher, Waltham, USA). Correctly inserted genes (determined by sequencing) were selected for further expression.

Text S2: Proteins identification

Recombinant proteins were identified after SDS-PAGE separation. The band at the expected molecular weight was cut from the gel and reduced with 100 µL of 0.15% (w/v) D-Dithiothreitol (DDT) in 100 mM Ammonium bicarbonate (AmBic). After incubation at 56°C for 30 minutes, DDT was removed and 100 µL of 1% (w/v) Iodoacetamide in 100 mM AmBic was added to the sample. Incubation was performed for 30 min at room temperature in the dark. Iodoacetamide was removed from the sample with two washes with 200 µL 50 mM AmBic in ethanol. The gel pieces were dehydrated with 200 µL of acetonitrile (ACN) and after removal of the liquid phase, the gel pieces were completely dried. The dried gel pieces were incubated at 37°C overnight in 8 µL of trypsin (5ng/µL) and 15 µL 50mM AmBic. The next day, the liquid phase containing the peptides was recovered in a clean vial and peptides were further extracted by two washes with 35 µL 50% (v/v) ACN, 0.1% (v/v) Trifluoroacetic acid (TFA). The extracted peptides were dried under vacuum for 2 hours. Peptides were re-solubilized in 0.7 µL 50% (v/v) ACN, 0.1% (v/v) TFA and spotted on a stainless steel OptiTOF 384 well plate (AB Sciex, Framingham, USA). 0.7 µL of α-Cyano-4-hydroxycinnamic acid (CHCA) matrix at 7mg/mL in 50% (v/v) ACN, 0.1% (v/v) TFA were added. Samples were analysed using the AB Sciex 5800 MALDI-TOF/TOF (Sciex, Framingham, USA), a MS spectrum was acquired and the ten most intense peaks, excluding known contaminants, were fragmented. Identification was performed using an in-house MASCOT server with a database containing the theoretical amino acid sequence of the recombinant proteins and standard settings (B. Printz, K. Sergeant, S. Lutts, C. Guignard, J. Renaut, and JF. Hausman, J Proteome Res 12:5160–5179, 2013, doi:10.1021/pr400590d). Additional *de novo* sequencing was done to confirm the presence of any signal peptides.
